# FASN activity is important for the initial stages of the induction of senescence

**DOI:** 10.1038/s41419-019-1550-0

**Published:** 2019-04-08

**Authors:** Juan Fafián-Labora, Paula Carpintero-Fernández, Samuel James Davison Jordan, Tamanna Shikh-Bahaei, Sana Mohammad Abdullah, Midusa Mahenthiran, José Antonio Rodríguez-Navarro, Maria Victoria Niklison-Chirou, Ana O’Loghlen

**Affiliations:** 1 Epigenetics and Cellular Senescence Group, Blizard Institute, Barts and The London School of Medicine and Dentistry, Queen Mary University of London, 4 Newark Street, London, E1 2AT UK; 2 Centre for Genomics and Child Health, Blizard Institute, Barts and the London School of Medicine and Dentistry, Queen Mary University of London, London, E1 2AT UK; 3 Instituto Ramón y Cajal de Investigaciones Sanitarias, Neurobiología-Investigación. Hospital Ramón y Cajal, 28034 Madrid, Spain

## Abstract

Senescent cells accumulate in several tissues during ageing and contribute to several pathological processes such as ageing and cancer. Senescence induction is a complex process not well defined yet and is characterized by a series of molecular changes acquired after an initial growth arrest. We found that fatty acid synthase (FASN) levels increase during the induction of senescence in mouse hepatic stellate cells and human primary fibroblasts. Importantly, we also observed a significant increase in FASN levels during ageing in mouse liver tissues. To probe the central role of FASN in senescence induction, we used a small-molecule inhibitor of FASN activity, C75. We found that C75 treatment prevented the induction of senescence in mouse and human senescent cells. Importantly, C75 also reduced the expression of the signature SASP factors interleukin 1α (IL-1α), IL-1β and IL-6, and suppressed the secretion of small extracellular vesicles. These findings were confirmed using a shRNA targeting FASN. In addition, we find that FASN inhibition induces metabolic changes in senescent cells. Our work underscores the importance of C75 as a pharmacological inhibitor for reducing the impact of senescent cell accumulation.

## Introduction

Accumulation of senescent cells in different tissues during ageing has been extensively reported, especially in cardiovascular and other age-related diseases^1^. Senescence is a process primarily characterized by a stable cell proliferation arrest and development of a secretory state known as senescence-associated secretory phenotype (SASP)^2^. SASP factors are mainly composed by cytokines, chemokines and growth factors. Senescent cells are able to affect adjacent cells through SASP factor activation of various cell-surface receptors and a resulting intracellular signal transduction^3,4^. As part of the SASP, senescent cells also secrete extracellular vesicles (EVs)^5,6^. Importantly, it was shown that the protein p53 is responsible for the regulation of EVs secretion^7^.

A key characteristic of senescent cells is their high metabolic activity^8^. Increased glycolysis and redox homoeostasis has been frequently detected in vitro in senescent cells, with decreased fatty acid oxidation^9^. Several signals and stressors can trigger senescence via accumulation of the protein p53, which consequently activates the cell cycle inhibitor p21^CIP^^10^. p53 belongs to a small family of tumour suppressor proteins known as the p53 family^11,12^. This family regulates several pathways in cells, the most well-known being apoptosis, cell cycle and senescence^2,11^.

p53 regulates glycolysis, oxidative phosphorylation and amino acid metabolism in cancer cells^13–15^, and also modulates metabolic adaptation in senescence cells^16^. Glycolysis and oxidative phosphorylation are the two major metabolic pathways involved in regulating most cellular activities. Although glycolysis and its metabolites have been widely studied in the context of senescence and cancer, fewer studies have been conducted on the role of lipid metabolism in inducing replicative senescence^8^. It is of note that p53 activation has been linked to alterations in fatty acid metabolism^17^ but the role of lipid synthesis in regulating senescence induction has been understudied.

Lipid synthesis is an important metabolic process in cells. Lipids are essential for the production of fatty acids, phospholipids, sterols and sphingolipids^18^. Fatty acids can be derived from two sources: exogenous sources or de novo fatty acid synthesis (FAS). Normal cells rely on exogenous fatty acids, whereas tumour cells and senescent cells use de novo synthesis of fatty acids^19^. The FAS pathway produces long-chain fatty acids, using the enzyme fatty acid synthase (FASN) to combine acetyl-CoA produced from glycolysis with malonyl-CoA. FASN plays a major role in FAS and has been shown to be upregulated in different cancer cells^20^. However, limited evidence has implicated FASN pathway in cellular senescence^21^.

To understand the link between FAS and cellular senescence, we decided to study the role of FASN activity at the initial phases of senescence activation in both mouse and human cells. We focused on the initial phase of senescence activation in order to identify future therapies that would prevent the activation of a full senescent programme. For this, we took advantage of a well-documented FASN inhibitor, C75. C75 has been largely used as a tool for studying FAS role in metabolic disorders, senescence and cancer^22^. In addition, we confirmed our findings using a previously characterized short hairpin RNA (shRNA) targeting *FASN* (shFASN)^23^. We next assessed the role of FASN during the initial stages of activation of the senescence programme. We found increased levels of *FASN* mRNA during the induction of senescence. Treatment of senescent cells with C75 and shFASN prevented the induction of senescence. C75 and shFASN also reduced the secretion of SASP factors interleukin 1α (IL-1α), IL-1β and IL-6, and suppressed the secretion of EVs. We found that FASN inhibition also induces metabolic changes in senescent cells.

Senescent cells accumulate during ageing in degenerative tissues, so drugs that can control SASP activity have the potential of improving several age-related diseases. The results from this study support the use of C75 as a novel therapeutic agent for reducing the effect of the activation of senescence on different age-associated diseases.

## Results

### p53 endogenous expression progressively induces senescence in mouse hepatic stellate cells

**Fig. 1:**
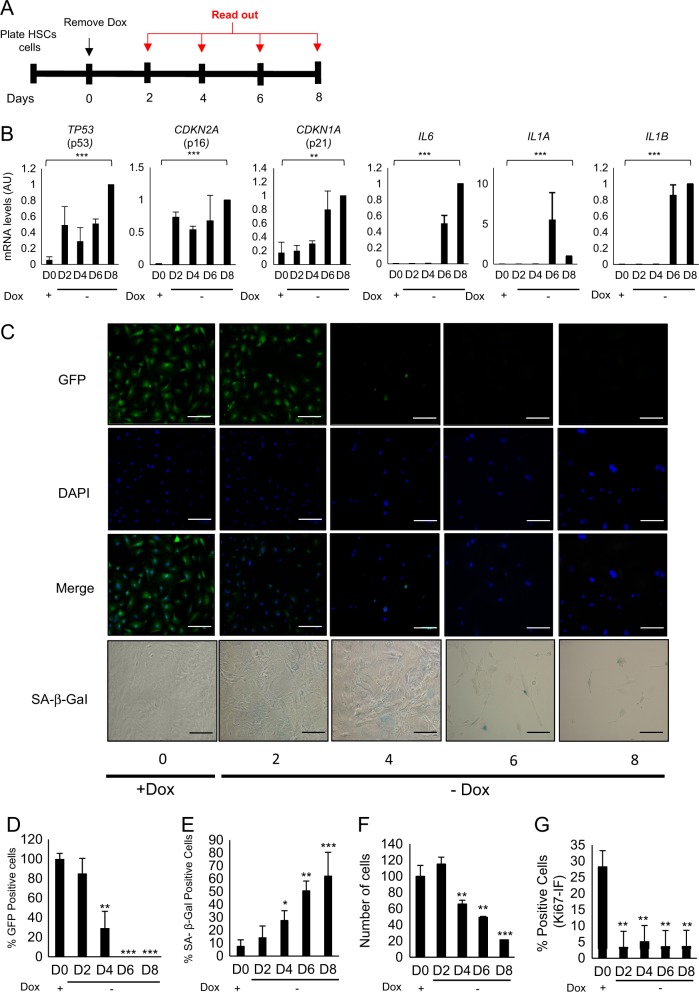
Evaluation of senescence induction in hepatic stellate cells (HSCs). **a** Schematic representation of the workflow followed for the senescence induction in HSCs. **b** Senescence was induced by doxycycline (Dox) withdrawal in HSCs at different days (0, 2, 4, 6 and 8 days). The expression levels of the senescence markers *TP53*, *CDKN2A*, *CDKN1A*, *IL6*, *IL1A* and *IL1B* were analysed by RT-PCR. Data represent the mean ± SEM of three independent experiments. **P < 0.001; ***P < 0.0001. **c** Representative images of GFP (green), DAPI (blue) and senescence-associated β-galactosidase (SA-β-gal) activity in control HSCs cells and after 0, 2, 4, 6 and 8 days of Dox removal. SA-β-Gal staining was assessed by light microscopy, and GFP and DAPI staining by fluorescent microscopy. Scale bars: 100 μm. **d**–**g** Quantification of different biomarkers of senescence in control HSCs and after 2, 4, 6 and 8 days of Dox withdrawal. Quantification of the following: **d** percentage of GFP-positive cells, **e** percentage SA-β-gal-positive cells, **f** relative cell numbers and **g** percentage of Ki67-positive cells. The graphs represent the mean ± SEM of three independent experiments. Two-tailed Student’s *t*-test was used to calculate the significance and it was represented as follows: *P < 0.05; **P < 0.001; ***P < 0.0001

In order to investigate the metabolic adaptation during the activation of senescence, we took advantage of hepatic stellate cells (HSCs) derived from adult mouse liver, previously described by Lujambio et al.^24^. HSCs were cultured in the presence of doxycycline (Dox) inducing the expression of a green fluorescent protein (GFP)-tagged shRNA targeting *TP53* (*GFP-shp53*) in a reverse tetracycline-controlled transactivator transgene vector (Supplementary Fig. S1A). HSCs were withdrawn of Dox and collected at different time points, allowing the endogenous expression of p53 (Fig. 1a). The endogenous expression of *TP53* and other senescence markers (*CDKN2A*, *CDKN1A*, *IL6*, *IL1A* and *IL1B*) were monitored by reverse transcription-PCR (RT-PCR) (Fig. 1b). We found a time-dependent increase in *TP53* mRNA level expression upon Dox removal. We also found increased expression of *CDKN2A* (encoding p16^INK4A^) and *CDKN1A* (encoding p21^CIP^), genes important in inducing cell cycle arrest and senescence^25^, after Dox was withdrawn (Fig. 1b). Notably, we observed increased transcription of *IL6*, *IL1A* and *IL1B*, key mediators of the proinflammatory elements of SASP^26^ (Fig. 1b). In the presence of Dox HSCs express high levels of GFP and after Dox withdrawal GFP expression was significantly reduced (Fig 1c, d). Indeed, HSCs after Dox removal showed increased SA-β-gal (senescence-associated β-galactosidase) activity (Fig. 1c, e) with reduced cell proliferation, measured by cell number by 4′,6-diamidino-2-phenylindole (DAPI) and quantifying cells positive for Ki67 staining (Fig. 1c, f, g). Overall, these results show that after Dox removal HSCs undergo a cell cycle arrest and express different markers of senescence.

### FASN activity is essential for mitochondria energy in senescent cells

**Fig. 2:**
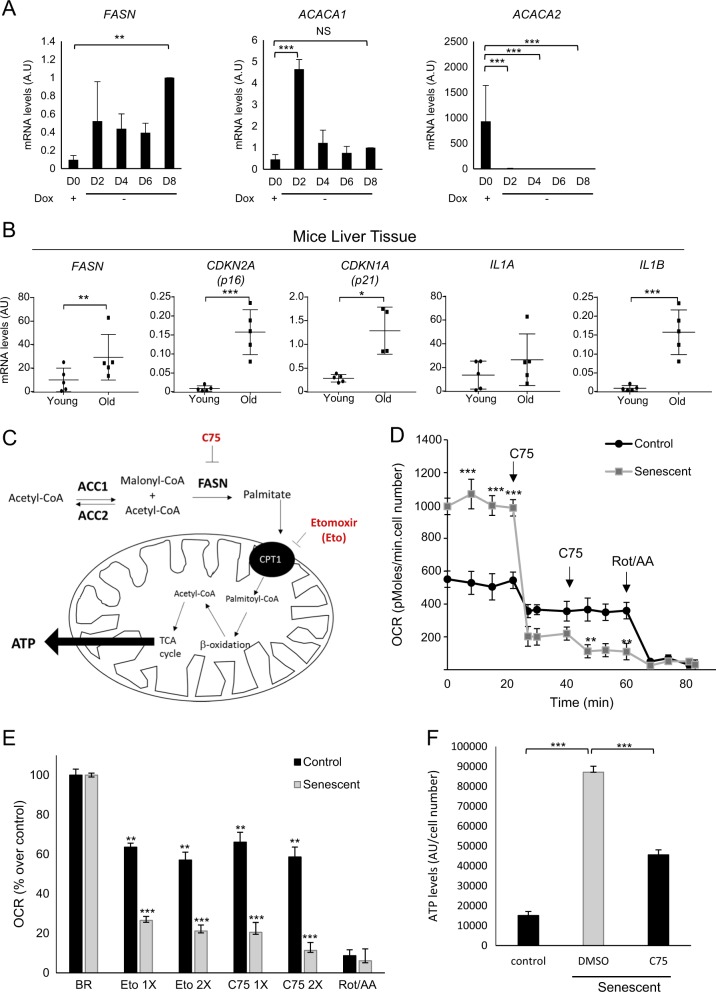
Fatty acid synthase pathway is important for the induction of senescent in HSCs. **a** Relative mRNA levels of fatty acid synthase pathway genes (*FASN*, *ACACA1* and *ACACA2*) in HSCs at different days after senescence induction by Dox depletion (0, 2, 4, 6 and 8 days). Data represent the mean ± SEM of three independent experiments. **P < 0.001; ***P < 0.0001; NS: not statistically significant. **b** mRNA levels are shown for *FASN*, *CDKN2A*, *CDKN1A*, *IL1A* and *IL1B* in livers taken from C57BL/6J female mice from young (4 months) and old (25 months) mice. Data represent the mean ± SEM of three independent experiments. *P < 0.01; **P < 0.001; ***P < 0.0001. **c** Schematic representation of the FASN pathway for ATP generation. ACC (acetyl-CoA carboxylase) transforms acetyl-CoA to malonyl-CoA; FASN (fatty acid synthase) catalyses the conversion of malonyl-CoA with acetyl-CoA to generate palmitate. Palmitate is then introduced into the mitochondria by the transporter CPT-1 (carnitine palmitoyltransferase 1). Next, palmitate goes through β-oxidation to generate acetyl-CoA, which gives substrate to the TCA cycle and generates ATP. **d**, **e** Seahorse Bioscience XF-24 extracellular flux analyser was used to measure oxygen consumption rate (OCR) (pMoles/min/cell number), in control and senescent HSCs cells. **d** OCR was measured in control and after 8 days of senescence induction in HSCs cells. OCR in response to C75 (10 μM) and a mix of rotenone (2 μM)/antimycin A (1 μM) (Rot/AA) was recorded to construct functional bioenergetic profiles. A minimum of five different samples was analysed for each group. **P < 0.001; ***P < 0.0001, unpaired two-sided *t*-test. Data from one representative experiment out of three is shown. **e** Data are represented as the % of the basal OCR after addition of different concentrations of C75 10 μM (1X) and 20 μM (2X), and etomoxir (Eto) 50 μM (1X) and 100 μM (2X). Data represent the mean ± SEM of three independent experiments. **P < 0.001; ***P < 0.0001. **f** ATP levels in HSCs control cells and after 8 days of senescence induction by Dox depletion. The graph represents the mean ± SEM of two independent experiments. ***P < 0.0001

p53 regulates the expression of several target genes involved in different metabolic pathways^13,14,15^. We explored whether senescence induction in HSCs was affecting lipogenesis. To this end, we measured changes in the FASN pathway by detecting mRNA levels of *FASN* and Acetyl-CoA carboxylase enzymes (*ACACA1* and *ACACA2*) (Fig. 2a). After removing Dox to induce senescence, we observed significant *FASN* mRNA upregulation with a significant reduction of *ACACA2*. *ACACA1* mRNA levels reached a peak at day 2 without Dox, after which it drops to basal levels.

To determine whether *FASN* increased mRNA levels in senescence correlated during ageing in vivo, we extracted RNA from C57BL/6J mice liver of 4 (young) and 25 (old) months. RT-PCR analysis of the livers from aged mice revealed elevated levels of *FASN* mRNA as compared with young mice (Fig. 2b). Importantly, we also observed increased levels of several well-established biomarkers of senescence such as *CDKN2A*, *CDKN1A*, *IL1A* and *IL1B* in livers from aged mice, suggesting increased senescence in vivo (Fig. 2b). Together, these data indicate that *FASN* mRNA levels increase during ageing in vivo.

As the FASN pathway plays a central role in mitochondrial energy metabolism^27^ (Fig. 2c), we next measured the oxygen consumption rate (OCR) in both control and senescent HSCs after 8 days of Dox withdrawal. As expected, senescent HSCs showed a greater increase in OCR than the control (Fig. 2d). These data are in agreement with previous reports showing that senescent cells have a marked increase in mitochondrial activity^6,28,29^. To assess whether this increase in OCR during senescence was due to FASN activity, we used increasing concentrations of C75. An 80% OCR reduction was observed in senescent HSCs after C75 treatment (Fig. 2d, e). Importantly, only a 20% OCR reduction was observed in control after C75 treatment. Next, we inhibited fatty acid oxidation in HSCs with increasing concentrations of etomoxir (Eto), an irreversible inactivator of the carnitine palmitoyltransferase-1 (CPT-1) transporter^30^. Eto reduced senescent cell OCR by almost 80% but only reduced control OCR by 35% (Fig. 2e). A combination of 2 μM rotenone and 1 μM antimycin A (mitochondria complex I and III inhibitors, respectively) was added to the cells, to shut down mitochondrial respiration as a positive control for OCR reduction (Fig. 2d, e). Collectively these data suggest that FASN activity is important for the mitochondrial energy metabolism during senescence.

It is well documented that senescent cells have an increase in mitochondria number and mitochondria hyperfunction^28^. Therefore, we measured mitochondria membrane potential and reactive oxygen species (ROS) in control and senescent HSCs with or without C75 (Supplementary Fig. S1B, C). As expected, we observed significant mitochondrial hyperpolarization in senescent cells with increased ROS, measured by quantifying hydrogen peroxide production. Importantly, C75 treatment was able to inhibit mitochondria hyperpolarization and ROS production (Supplementary Fig. S1B, C). To determine whether the mitochondria hyperpolarization and ROS production could be due to an increase in the number of mitochondria in our model, we measured COXIV, a mitochondrial marker, by western blotting (Supplementary Fig. S1D). In fact, we detect a clear increase in COXIV in senescent cells as compared with control as previously described^28^. Importantly, C75 was able to reduce the mitochondria mass at similar levels to control (Supplementary Fig. S1D). As ATP levels in the cells mainly depend on mitochondrial activity^31^, we next measured ATP levels in senescent cells after 6 days of Dox withdrawal vs. control. We found that ATP levels in senescent cells increased significantly in comparison with control (Fig. 2f). Importantly, strong reduction in ATP levels was observed after C75 treatment in senescent cells (Fig. 2f). These data are in agreement with our previous findings that mitochondria in senescent cells exhibited a marked increase in basal oxygen consumption, which was greatly reduced following treatment with C75 (Fig. 2d). In addition, C75 is able to prevent mitochondria dysfunctional during senescence with enhanced levels of ROS. Next, to validate these data in an in vivo system, we quantified the levels of mRNA of peroxisome proliferator-activated receptor-γ coactivator 1-α (*PGC1A*) as a marker of mitochondria biogenesis in ageing mice. We detect a significant increase of *PGC1A* transcript in the livers of old mice as compared with that of young mice (Supplementary Fig. S1E), suggesting that old mice have an increased mitochondria number and biogenesis. In agreement with these results, a significant increase in mitochondria basal respiration was observed in mitochondria isolated from the liver of old mice as compared with that of young mice (Supplementary Fig. S1F). Collectively, these data suggest that FASN inhibition prevents mitochondria transformation arising during senescence.

### The FASN inhibitor C75 affects mTOR activity and reverses growth arrest in senescent cells

**Fig. 3:**
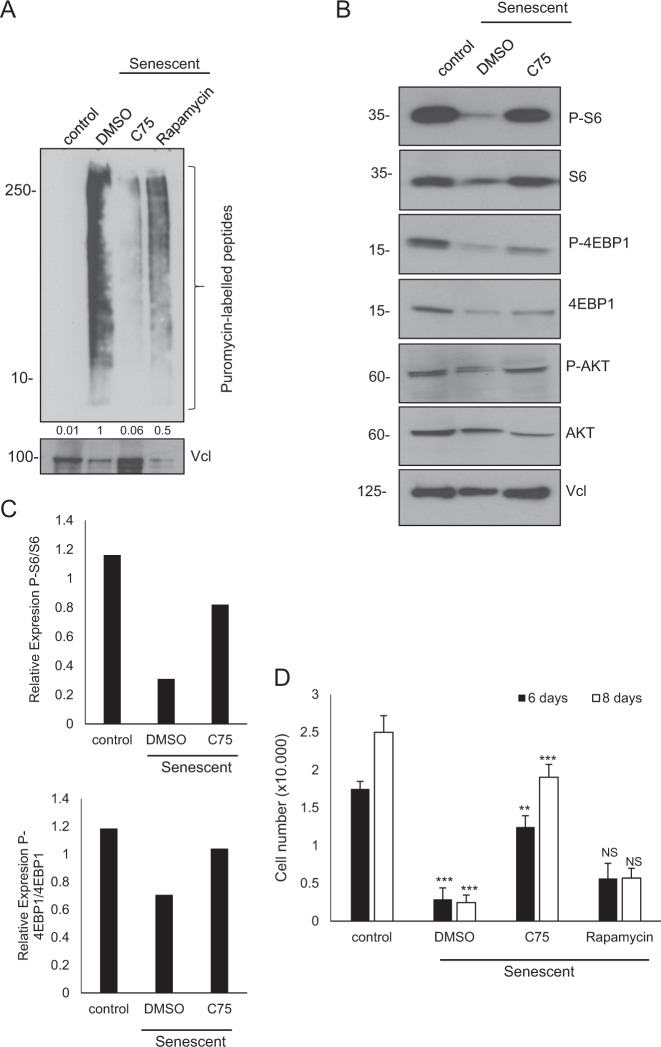
C75 treatment reduces global protein synthesis in senescent HSCs. **a** Control, senescent (6 days Dox removal) and senescent cells treated with 10 μM C75 or 1 μM rapamycin were treated for 1 h with 2.5 μg/mL puromycin. Western blotting of protein extract was probed with an anti-puromycin antibody. Vinculin (Vcl) was used as a loading control. Band densitometry is shown below each lane and normalized to the senescent sample as a value of 1. **b** Western blotting was performed for S6K, p-S6K(Ser240/244), 4E-BP1, p-4E-BP1(Ser65), AKT and p-AKT (Ser473) in control, senescent and senescent with 10 μM C75. Vcl was used as a loading control. **c** Quantification of p-S6K/S6K and p-4E-BP1/4E-BP1 blottings. **d** Cell number was assessed at days 6 or 8 days after senescence induction by Dox withdrawal. Senescent cells were treated with vehicle or 10 μM C75 or 1 μM rapamycin. **P < 0.001; ***P < 0.0001; NS: not statistically significant

The mammalian target of rapamycin (mTOR) senses signals from various growth factors and nutrients, and regulates several pathways involved in protein and lipid synthesis^32^. mTOR is activated in cellular senescence, promoting protein synthesis^33^. We found that senescent HSCs displayed a significant increase in the amount of puromycin-labelled peptides compared with control, indicating that global protein synthesis^34^ is increased in senescent cells and suggesting an increase in mTOR activity (Fig. 3a). Importantly, a significant reduction of protein synthesis of 90% was found in senescent cells treated with C75 (Fig. 3a). We used 1 μM rapamycin, a mTOR inhibitor, as a positive control for mTOR activity and could observe a 50% reduction in protein synthesis in senescent cells treated with rapamycin. Our results suggest that senescent cells upregulate global rate of protein synthesis, and that FASN inhibition is able to restore the normal ratio of protein synthesis in the cells.

To confirm the implication of mTOR, we analysed mTOR signalling after 6 days of senescence induction and we compared this with control. To our surprise, mTOR activity was repressed during senescence as shown by a decrease in the phosphorylation levels of some of its downstream targets: S6, 4EBP1 and AKT (Fig. 3b, c). However, it was recently reported that mTOR is quickly activated in senescence but after 72 h this signal is inhibited by a negative feedback loop^35^. Remarkably, senescent cells treated with C75 were able to restore mTOR signalling at levels similar to control (Fig. 3b, c).

To validate the role of mTOR during ageing in an in vivo system, we measured in aged mouse liver *EEF2* and *MAPKAPK2* transcripts, which are two canonical targets of mTOR^35^. We observed a significant increase in *EEF2* and *MAPKAPK2* mRNA levels in the livers of old mice (Supplementary Fig. S1G), suggesting an upregulation of mTOR during ageing as previously described^36^.

mTOR inhibition has been shown to inhibit the SASP, but there is controversy whether it reverses the proliferation arrest characteristic of senescence^35^. Therefore, we measured cell number after 6 and 8 days of senescence induction (Fig. 3d). We observed a significant cell number reduction after the induction of senescence and treatment with C75 rescued the growth arrest of senescent HSCs (Fig. 3d). However, mTOR inhibition with 1 μM rapamycin did not rescue the cell cycle arrest induced in senescence (Fig. 3d).

### FASN inhibition prevents the initiation of senescence induction in HSCs

**Fig. 4:**
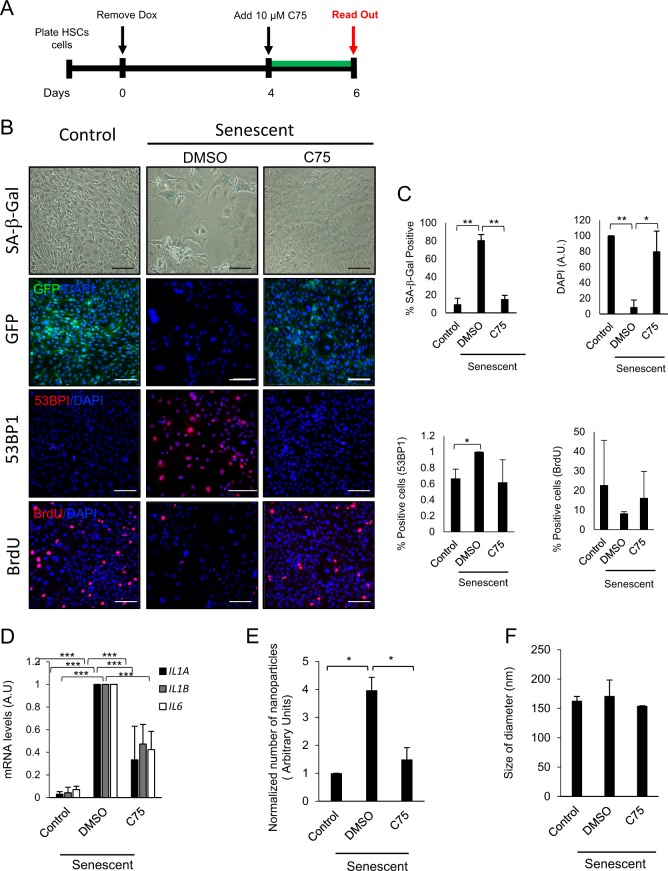
The FASN inhibitor, C75, prevents the induction of cellular senescence. **a** Schematic representation of the experimental settings to determine the role of FASN pathway in preventing the induction of senescence in HSCs. After 4 days of Dox withdrawal, 10 μM C75 was added to HSCs. **b** Representative images of control, senescent and senescent with 10 μM C75 treatment in HSCs. SA-β-Gal staining was assessed by light microscopy. GFP (green), DAPI (blue), 53BPI and BrdU (red) staining was assessed and quantified using the automated fluorescent microscope InCell 2200. Scale bar: 100 μm. **c** Quantification of the percentage of HSC staining positive for SA-β-gal, GFP, 53BP1 and BrdU incorporation in control and senescent HSCs with or without 10 μM C75 treatment. Data represent the mean ± SEM of three independent experiments. **d** Relative expression of *IL1A*, *IL1B* and *IL6* in control, senescent and senescent plus 10 μM C75 HSCs. **e** Extracellular vesicles (EVs) released in control, senescent and senescent plus 10 μM C75 HSCs were measured using Nanoparticle Tracking Analysis (NTA). **f** Evaluation of the size distribution of the EVs released by HSCs in control, senescent and senescent plus 10 μM C75 treatment. Data represent the mean ± SEM of two independent experiments. Two-tailed Student’s *t*-test was used to calculate the significance and it was represented as follows: *P < 0.05; **P < 0.001; ***P < 0.0001

To assess the relevance of FASN activity in the establishment of the senescent programme, we treated HSCs with C75 before the establishment of senescence after 4 days of Dox withdrawal (Fig. 4a). Next, we evaluated whether senescence was established at day 6 of Dox removal by measuring several senescence markers. As shown in Fig. 4b, after 6 days of Dox withdrawal, HSCs displayed a senescent phenotype. Indeed, HSCs show a significant increase in SA-β-gal activity and in the percentage of cells expressing the tumour suppressor p53-binding protein 1 (53BP1), an indication of DNA damage and significant reduction in GFP expression (Fig. 4b, c). Thus, bromodeoxyuridine (BrdU) immunostaining revealed cell proliferation arrest after Dox withdrawal compared with control (Fig. 4b, c). Importantly, C75 treatment of HSCs showed a marked ability to prevent the activation of the senescent phenotype indicated by higher levels of BrdU staining and low SA-β-gal activity (Fig. 4b, c). In agreement with these data, p53, p16 and p21 RNA and protein levels were also upregulated after senescence induction and significantly reduced after C75 treatment (Supplementary Fig. S2A, B, C). Importantly, C75 treatment did not induce apoptosis in HSCs senescent cells (Supplementary Fig. S2D).

Senescent cells secrete large amounts of molecules as part of the SASP, which induce changes in the microenvironment^26^. We next evaluated whether C75 could reduce the secretion of the SASP factors by RT-PCR measuring the mRNA levels of *IL1A*, *IL1B* and *IL6* in senescent cells. Figure 4d shows that senescent cells treated with C75 significantly reduced *IL1A*, *IL1B* and *IL6* mRNA levels in HSCs.

It was reported that cancer cells upregulate FAO and the β-oxidation pathway to achieve the extra energy demand, and it has been suggested that these two processes are linked together^9,37^. Therefore, we used the β-oxidation pathway inhibitor Eto^38^ and we observed a marked senescence-induction inhibition indicated by a significant reduction in SA-β-Gal staining when senescent HSCs were treated with Eto (Supplementary Fig. S3A, B). Indeed, RNA levels for the cell cycle inhibitors *TP53*, *CDKN2A* and *CDKN1A* were significantly downregulated upon Eto treatment (Supplementary Fig. S3C). These results further support the idea that fatty acid metabolism (synthesis and oxidation) play a central role in the induction of senescence. We therefore conclude that blocking lipid metabolism with two independent inhibitors prevents the activation of senescence in HSCs.

A key and novel aspect of p53 function is the regulation of EVs secretion, which is an important process involved in cell–cell communication^7^. Our analysis revealed that FASN activity regulates p53 expression and, due to this, we wondered whether FASN activity could regulate EVs secretion. To this end, we induced senescence in HSCs and then we treated the cells with and without C75. We observed that senescent cells secrete high numbers of EVs as described before^6^, but after treatment with C75 we observed a statistically significant decreased in the secretion of EV compared with senescent cells (Fig. 4e). Moreover, no differences in EVs size distribution between control, senescent and senescence plus C75 was observed (Fig. 4f).

**Fig. 5:**
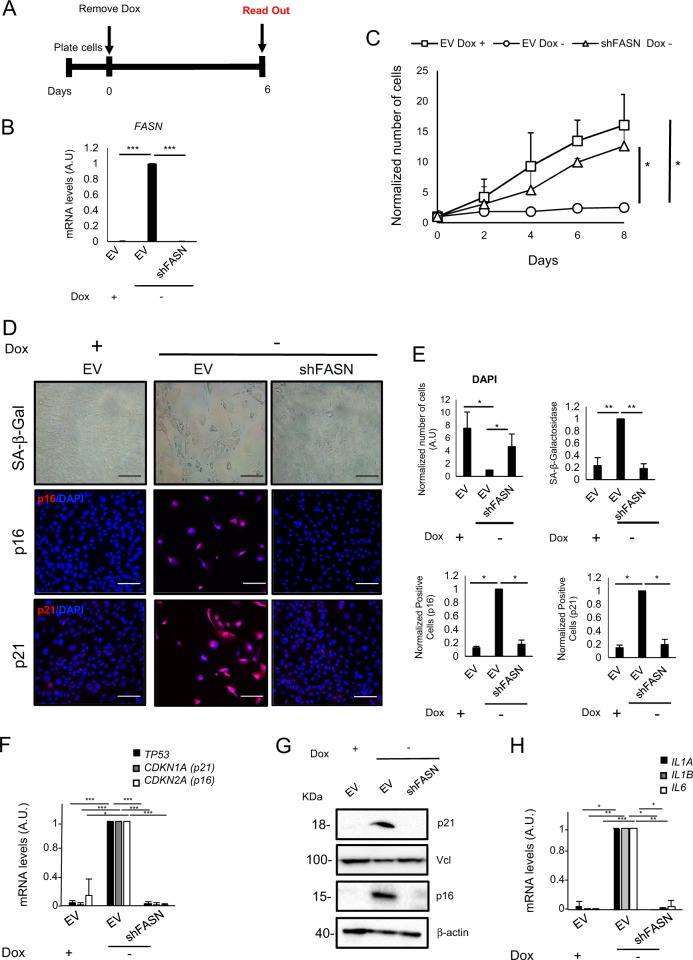
FASN knockdown prevents cellular senescence induction in hepatic stellate cells. **a** Schematic representation of the experimental settings to determinate the role of FASN knockdown using a short hairpin RNA targeting FASN (shFASN). HSCs cells were infected with empty vector control (EV) or shFASN and then selected with puromycin. After selection, cells were plated and Dox was removed to induced senescence for 6 days. **b** Relative expression of levels of *FASN* transcript in empty vector plus (EV Dox+; control HSCs), empty vector without doxycycline (EV Dox−; senescent HSCs) and shFASN without doxycycline (shFASN Dox−; senescent + shFASN) infected HSCs cells. Data represent the mean ± SEM of three independent experiments. ***P < 0.0001. **c** Growth curve of control (EV Dox+), senescent (EV Dox−) and senescent + shFASN (shFASN Dox−) HSCs expressed as number of cells at different time points of Dox withdrawal. Data represent the mean ± SEM of three independent experiments. *P < 0.01. **d** Representative images of control, senescent and senescent + shFASN stained with p16 and p21 antibodies. SA-β-Gal staining was assessed by light microscopy. p16 and p21 (red), and DAPI (blue) staining was assessed and quantified using the automated fluorescent microscope InCell 2200. Scale bar: 100 μm. **e** Quantification of cell number by DAPI and the percentage of cells staining positive for p16, p21 and SA-β-Gal in control, senescent and senescent + shFASN HSCs. Data represent the mean ± SD of three independent experiments. *P < 0.01. **f** Relative mRNA expression measured by RT-PCR of *TP53*, *CDKN1A* and *CDKN2A* in HSCs. **g** Western blotting was performed for p16 and p21 in control, senescent and senescent + shFASN HSCs. β-Actin and vinculin (Vcl) were used as a loading control. **h** Relative mRNA expression of *IL1A*, *IL1B* and *IL6* in control, senescent and senescent + shFASN HSCs. *P < 0.01; **P < 0.001; ***P < 0.0001

To determine the specificity of C75 in inhibiting FASN, we silenced FASN expression in HSCs using a previously validated lentiviral vector encoding a FASN-specific shRNAs (shFASN)^23^, which reduced *FASN* levels (Fig. 5a, b). Importantly, FASN knockdown in senescent HSCs prevented the senescent-induced cell cycle arrest (Fig. 5c–e), in addition to preventing the upregulation of different markers of senescence such as SA-β-gal activity, p53, p16 and p21 (Fig. 5d–g). Notably, FASN knockdown also inhibit the increase in the expression of mRNA levels of SASP factors *IL1A*, *IL1B* and *IL6* in HSCs (Fig. 5h). These data show that FASN activity is important for the induction of senescence in HSCs.

### C75 is able to prevent the induction of senescence in human primary fibroblasts

**Fig. 6:**
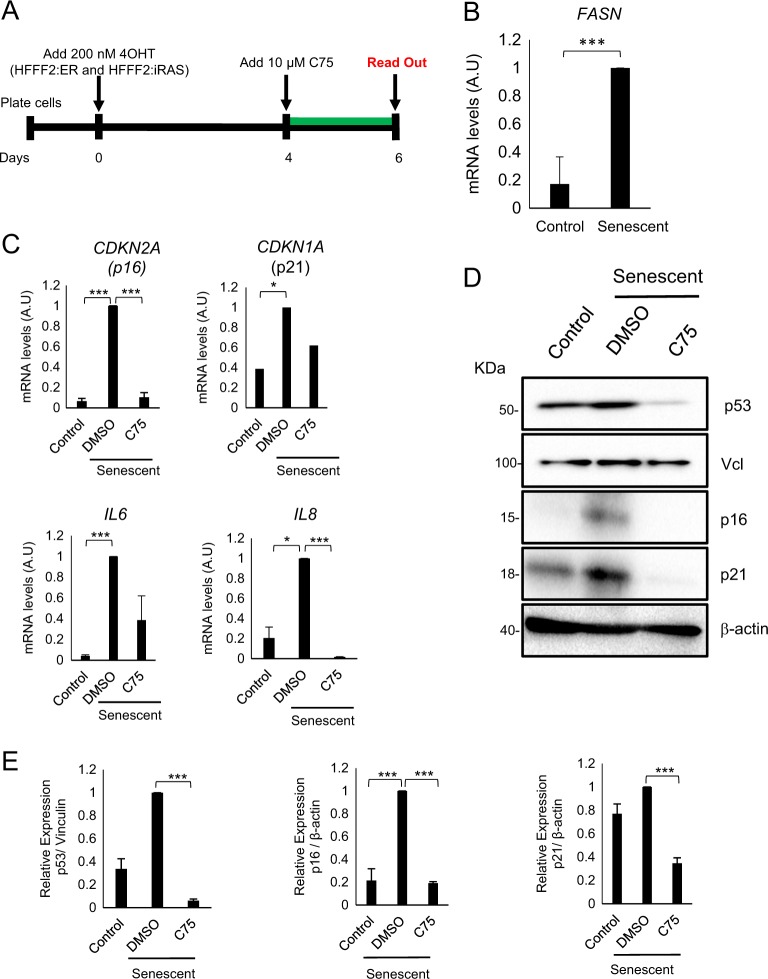
The C75 inhibitor blocks senescence in human primary fibroblasts. **a** Schematic representation of the experimental settings to determinate the role of FASN pathway in human primary fibroblast. Human primary foreskin fibroblasts (HFFF2) were transduced with retroviruses expressing H-RAS^G12V^ or with empty vector control. After 4 days of senescent induction using 200 nM tamoxifen (4OHT), the cells were treated or not with 10 μM C75. **b** RT-PCR determining the mRNA levels of FASN in control and senescent human fibroblasts. **c** Relative mRNA levels of two cell cycle inhibitors, *CDKN2A* and *CDKN1A*, and two genes associated with SASP, *IL6* and *IL8*, were analysed in control, senescent and senescent plus 10 μM C75 treatment HFFF2 cells. Data represent the mean ± SEM of three independent experiments. **d** Western blottings of cellular lysates corresponding to cells transduced with control or with H-RAS^G12V^. Senescent cells were treated with DMSO or 10 μM C75. Expression of p53, p16 and p21 protein levels were analysed. β-Actin and vinculin (VcI) were used as a loading control. **e** Densitometry quantification for p53, p16 and p21 expression normalized with respect to vinculin or β-actin in HFFF2 expressing a control vector or H-RAS^G12V^. Senescent cells were treated with 10 μM C75 or DMSO. Two-tailed Student’s *t*-test was used to calculate the significance and it was represented as follows: *P < 0.05; ***P < 0.0001

In order to understand whether the mechanism underlying C75 prevention of cellular senescence initiation was conserved in human cells, we induced senescence in human primary foreskin fibroblasts (HFFF2) by a stable infection with a construct expressing the oncogene H-RAS^G12V^ (RAS) inducible by tamoxifen^39^ (Fig. 6a). First, we confirmed that *FASN* mRNA levels were indeed upregulated during senescence induced by RAS in human cells (Fig. 6b). Furthermore, RAS-induced senescent cells exhibited high RNA and protein levels of the senescent markers p53, p16 and p21 (Fig. 6c–e). Compared with control, RAS-induced senescence resulted in a strong increase in the levels of the secreted cytokines *IL6* and *IL8* (Fig. 6c). Significant inhibition of cell cycle inhibitors and SASP genes in RAS-induced senescent cells were observed after treatment with C75 (Fig. 6c–e).

Together, our data show that FASN activity is essential for the induction of senescence in human cells. In summary, our results support the idea that FASN has a novel role as a metabolic-controlling protein.

## Discussion

Cellular senescence is a well-defined phenotype characterized by a stable cell cycle arrest in response to different stress signals such as oncogene activation and DNA damage, and accompanied by a characteristic secretory phenotype of EVs and SASP^5,6,26,40^. Here we focused on elucidating the biological role of FASN in controlling the induction of cellular senescence.

FASN is the key enzyme involved in the regulation of de novo FAS^41^. Normal human tissues will use exogenous lipids for membrane synthesis, whereas de novo FAS is suppressed, and FASN expression is maintained at very low levels. We show here that FASN endogenous levels are upregulated during senescence in mouse and human cells. Numerous evidence suggest that cancer cells have deregulated lipid metabolism (de novo FAS and β-oxidation pathway)^41–43^, which resemble our findings.

We show here that FASN inhibition in mouse and human cells stops cellular senescence induction, by reducing mitochondrial energy levels, a finding that confirms and further extends the original description that senescent cells display high metabolic activity^44^.

FASN has a well-defined role in lipogenesis, by catalysing de novo biogenesis of fatty acids. New reports have given to FASN a central role in supporting cancer cell growth rather than functioning as an anabolic energy-storage pathway. Importantly, a recent study shows that FASN indirectly controls the mTOR, a central regulator of cellular metabolism^23,45^. Therefore, we set out to assess whether FASN regulates any aspect of mitochondria oxygen consumption in senescent cells and characterized the relationship between FASN inhibitor and cellular senescence induction. We induced senescence in mouse HSCs and assessed metabolic changes by measuring mitochondria respiration and ATP levels. Mitochondrial respiration is the most important generator of cellular energy under most circumstances^46^. It is a process of energy conversion of substrates into ATP. Importantly, we observed enhanced mitochondrial respiration with a robust increase in ATP levels in senescent cells. These results are in agreement with previous reports showing that RAS-induced senescent cells have increased OCR than control^47^. Overall, our study shows that FASN activity is important for mitochondrial bioenergetic generation in senescent cells.

This study unveils a role for FASN in controlling senescence establishment that contributes to explain the effect of FASN inhibitor, C75^27^. Importantly, C75 was reported to inhibit senescence in human diploid fibroblasts^21^, although it was unclear which senescent pathway was affected, whereas other reports show that C75 induce senescence in fibroblast^48^. Here we report that cellular senescence is inhibited in HSCs not only by treatment with C75 but also by FASN knockdown using a specific shRNA. Indeed, mouse and human cells treated with C75 inhibit cellular senescence induction and start to divide; they show reduced β-galactosidase staining and reduced p53 levels.

Rise of senescent cells in organs is considered to be the one of the hallmarks of ageing and therefore partially responsible for different age-associated diseases. Senescent cells can play a central role in this phenomenon, by secreting different SASP and EVs factors^6,40,49,50^. We show that C75 was able to inhibit the key mediators of the proinflammatory elements of the SASP and EVs secretion, possibly because we observed a dramatic reduction in p53 levels known to be the regulator of SASP and EVs^26^. Also, we observed increase expression of FASN and it was associated with increased levels in several biomarkers of senescence in liver tissue from aged mice. As senescent cells accumulate during ageing, causing cancer and several age-related pathologies, C75 could be a potential therapeutic route to increase the lifespan and delay age-related pathologies.

In summary, we show that FASN activity was vital for mitochondria respiration in senescent cells. Our results support the use of C75, as a drug for reducing the effect of different age-associated diseases.

## Materials and methods

### Cell culture

Mouse HSCs were a kind gift from Scott Lowe and were grown in 1 µg/ml of Dox. Human foreskin fibroblasts (HFFF2; 86031405) were purchased from Public Health England (UK). All cells were grown in high glucose Dulbecco’s modified Eagle’s medium (DMEM) with 10% fetal bovine serum and 1% antibiotic–antimycotic solution.

### RNA extraction, cDNA synthesis and RT-PCR

Cells grown in six-well plates or 10 cm dishes were washed with phosphate-buffered saline (PBS) and lysed directly into the culture dish using TRIzol Reagent (ThermoFisher). cDNA synthesis was performed using the High-Capacity cDNA Reverse Transcriptase Kit (ThermoFisher). RT-PCR reactions were performed using SYBR Green PCR Master Mix (Applied Biosystems) on a 7500 Fast System RealTime PCR cycler (Applied Biosystems). Primer sequences are listed in Supplementary Fig. S4.

### Stable gene expression

Stable retroviral and lentiviral expressions were performed as in previous studies^3^.

### β-Galactosidase staining

Cells were washed with PBS and fixed with 0.05% (w/v) glutaraldehyde (in PBS) for 15 min at room temperature (RT). Cells were washed a second time with PBS and incubated with 5-bromo 4-chloro-3-indolyl-beta-d-galacto-pyranoside solution (Promega) for 1 h at 37 °C. Cells were imaged using a light microscope (Nikon) at ×20 magnification and single representative images of each well were taken.

### Immunofluorescence staining and analyses

Cells grown in 96-well plates were washed with PBS and fixed in 4% paraformaldehyde for 15 min at RT. Cell were then washed in PBS twice before being permeabilized and blocked for 40 min with 0.2% Triton X-100 together with 1% bovine serum albumin and 0.2% gelatin fish (Sigma). For immunofluorescence (IF) staining, cells were incubated overnight with the primary antibody (Supplementary Fig. S5); in the case of BrdU, cells were treated with DNaseI and MgCl_2_. Cells were then washed in PBS and incubated 1 h with secondary antibody, DAPI (Sigma-Aldrich). IF images were acquired using IN Cell 2200 automated 991 microscope (GE) and the IN Cell 2200 Developer software version 1.8 (GE).

### Protein analysis by western blotting

Proteins from cells were lysed using the RIPA Buffer Lysis (ThermoFisher), separated in an SDS-polyacrylamide gel electrophoresis, transferred to a polyvinylidene difluoride membrane (Millipore) and probed with different antibodies (Supplementary Fig. S5).

### ATP levels

HSCs were depleted of Dox for 6 days. Cells were trypsinized and centrifuged at 1000 r.p.m. Collected cells were suspended in 50 μL CellTiter-Glo Luminescent Cell Viability Assay solution (Promega, Madison, WI, USA) and incubated for 10 min. The luminescence intensity was measured using a Synergy HT Multi-Mode Microplate Reader. All the readings were expressed as rlu (relative luminescence unit)/cell number^51^.

### Measurement of cellular OCR

XF-24 cell culture microplates were coated with 3.4 mg/mL BD Cell-Tak^TM^ tissue adhesive solution (BD Bioscience 354240) according to the manufacturer’s instruction. Forty-three microlitres of the Cell-Tak solution was added to each well of a XF-24 cell culture plate and incubated for 20 min at RT. Mouse HSCs were plated at densities of 30,000 cells per well in a Seahorse XF-24 plates pre-coated with Cell-TAK on the same day of the analysis. Microplates containing the cell suspension were centrifuged at 700 × *g* for 5 min and then incubated at 37 °C for at least 1 h, to allow attachment. Before measurements, the growth medium was replaced with 600 μl assay medium (Seahorse Bioscience), a low buffered DMEM containing no bicarbonate, and incubated for 45 min in a 37 °C non-CO_2_ incubator. Basal OCR was determined using XF-24 Extracellular Flux analyser. The different drugs were added automatically during measurement, after establishing the baseline of OCRs. We measured the OCR in response to sequential treatment with the irreversible inhibitor of CPT-1, Eto at 50 µM and 100 μM, an inhibitor of FASN, C75 at 10 μM and 20 μM, and with a mix of the electron transport chain inhibitors rotenone (2 μM) and antimycin A (1 μM)^51^.

### Nanoparticle tracking analysis

The NanoSight LM10 (Malvern Instruments) was calibrated using Silica Microspheres beads (Polyscience). Samples were diluted in PBS in order to obtain a particle number between 10^8^–10^9^ particles. At least three repeated measurements of 60 s were taken per each individual sample and the mean value was used to determine particle number. The movement of each particle in the field of view was measured to generate the average displacement of each particle per unit time, which was calculated using the NTA 3.0 software^52^.

### Newly synthesized proteins

HSCs cells were plated and treated as indicated. After 6 days, control, senescent, senescent + C75 and senescent + 1 μM rapamycin cells were treated for 1 h with 2.5 μg/ml puromycin. Cells were collect with RIPA buffer. The incorporation of puromycin in the newly synthesized proteins was assessed by western blotting with an anti-puromycin antibody^34^.

### Liver mice tissue

Livers were taken from female C57BL/6J mice. All mice (4- and 25-months old) were maintained in the same housing with identical environmental conditions. Tissues were provided by the Tissue Bank provider ShARMUK.

### Isolation of liver mitochondrial

Livers were taken from female C57BL/6J mice. All mice (4- and 25-months old) were maintained in the same housing with identical environmental conditions. Mitochondria were isolated as previous described^53^.

### Apoptosis measurement

Apoptosis was quantified using the Annexin V, Alexa Fluor™ 647 conjugate (ThermoFisher Scientific, Catalogue number A23204). Briefly, cell medium and cell pellets were collected in a fluorescence-activated cell sorting (FACS) tube. Annexin V-647 (25 μg/ml) was added to the cells. The cells were incubated for 10 min in the darkness at RT. DAPI staining solution (1 μg/ml) was then added and FACS was carried out using a BD FACS CANTO II flow cytometer.

### Mitochondrial membrane potential

3,3′-dihexyloxacarbocyanine iodide (DiOC6) (Invitrogen, Catalogue number D273) staining was carried out as previously described^51^. Briefly, cells were washed once in FACS buffer and then resuspended in 300 μL of the same buffer containing 4 nM DiOC6. Cells were then incubated for 30 min at RT, followed by one wash with PBS. Cells were resuspended in PBS containing 200 ng/mL DAPI staining.

### Hydrogen peroxide assay (ROS)

Amplex Red Hydrogen Peroxide/Peroxidase Assay Kit (ThermoFisher Scientific) was used to determine the amount of hydrogen peroxide (H_2_O_2_) present in cell samples as a marker for oxidative stress. Absorbance at 562 nm was measured using a Synergy HT Multi-Mode Microplate Reader.

### Statistical analyses

All results are expressed as mean values ± SD or ±SEM of at least three independent experiments, except when otherwise indicated. The unpaired Student’s *t*-test and one-way analysis of variance were used to compare and identify statistically significant differences.

## Supplementary information

Figure S1, Figure S2, Figure S3, Figure S4, Figure S5

suplementary figure legends

## References

[1] Campisi J (2005). Senescent cells, tumor suppression, and organismal aging: good citizens, bad neighbors. Cell.

[2] Munoz-Espin D, Serrano M (2014). Cellular senescence: from physiology to pathology. Nat. Rev. Mol. Cell Biol..

[3] Rapisarda V (2017). Integrin beta 3 regulates cellular senescence by activating the TGF-beta pathway. Cell Rep..

[4] Hoare M (2016). NOTCH1 mediates a switch between two distinct secretomes during senescence. Nat. Cell Biol..

[5] Takahashi A (2017). Exosomes maintain cellular homeostasis by excreting harmful DNA from cells. Nat. Commun..

[6] Borghesan, M. et al. Exosomes are key regulators of non-cell autonomous communication in senescence. *bioRxiv*10.1101/356238 (2018).

[7] Yu X, Harris SL, Levine AJ (2006). The regulation of exosome secretion: a novel function of the p53 protein. Cancer Res..

[8] Quijano C (2012). Oncogene-induced senescence results in marked metabolic and bioenergetic alterations. Cell Cycle.

[9] Wiley CD, Campisi J (2016). From ancient pathways to aging cells-connecting metabolism and cellular senescence. Cell. Metab..

[10] Campisi J (2013). Aging, cellular senescence, and cancer. Annu. Rev. Physiol..

[11] Allocati N, Di Ilio C, De Laurenzi V (2012). p63/p73 in the control of cell cycle and cell death. Exp. Cell Res..

[12] Kastenhuber ER, Lowe SW (2017). Putting p53 in context. Cell.

[13] Kawauchi K, Araki K, Tobiume K, Tanaka N (2008). p53 regulates glucose metabolism through an IKK-NF-kappaB pathway and inhibits cell transformation. Nat. Cell Biol..

[14] Hu W (2010). Glutaminase 2, a novel p53 target gene regulating energy metabolism and antioxidant function. Proc. Natl Acad. Sci. USA.

[15] Laezza C (2015). p53 regulates the mevalonate pathway in human glioblastoma multiforme. Cell Death Dis..

[16] Rufini A, Tucci P, Celardo I, Melino G (2013). Senescence and aging: the critical roles of p53. Oncogene.

[17] Ford JH (2010). Saturated fatty acid metabolism is key link between cell division, cancer, and senescence in cellular and whole organism aging. Age (Dordr).

[18] Currie E, Schulze A, Zechner R, Walther TC, Farese RV (2013). Cellular fatty acid metabolism and cancer. Cell. Metab..

[19] Medes G, Thomas A, Weinhouse S (1953). Metabolism of neoplastic tissue. IV. A study of lipid synthesis in neoplastic tissue slices in vitro. Cancer Res..

[20] Kuhajda FP (2000). Synthesis and antitumor activity of an inhibitor of fatty acid synthase. Proc. Natl Acad. Sci. USA.

[21] Kim YM (2010). Sterol regulatory element-binding protein (SREBP)-1-mediated lipogenesis is involved in cell senescence. J. Biol. Chem..

[22] Matsuo S, Yang WL, Aziz M, Kameoka S, Wang P (2014). Fatty acid synthase inhibitor C75 ameliorates experimental colitis. Mol. Med..

[23] Bruning U (2018). Impairment of angiogenesis by fatty acid synthase inhibition involves mTOR malonylation. Cell. Metab..

[24] Lujambio A (2013). Non-cell-autonomous tumor suppression by p53. Cell.

[25] Childs BG, Baker DJ, Kirkland JL, Campisi J, van Deursen JM (2014). Senescence and apoptosis: dueling or complementary cell fates?. EMBO Rep..

[26] Coppe JP (2008). Senescence-associated secretory phenotypes reveal cell-nonautonomous functions of oncogenic RAS and the p53 tumor suppressor. PLoS Biol..

[27] Landree LE (2004). C75, a fatty acid synthase inhibitor, modulates AMP-activated protein kinase to alter neuronal energy metabolism. J. Biol. Chem..

[28] Correia-Melo C (2016). Mitochondria are required for pro-ageing features of the senescent phenotype. EMBO J..

[29] Helman A (2016). p16(Ink4a)-induced senescence of pancreatic beta cells enhances insulin secretion. Nat. Med..

[30] Zhou W (2003). Fatty acid synthase inhibition triggers apoptosis during S phase in human cancer cells. Cancer Res..

[31] Salin K, Auer SK, Rey B, Selman C, Metcalfe NB (2015). Variation in the link between oxygen consumption and ATP production, and its relevance for animal performance. Proc. Biol. Sci..

[32] Menendez JA, Vellon L, Oliveras-Ferraros C, Cufi S, Vazquez-Martin A (2011). mTOR-regulated senescence and autophagy during reprogramming of somatic cells to pluripotency: a roadmap from energy metabolism to stem cell renewal and aging. Cell Cycle.

[33] Narita M (2011). Spatial coupling of mTOR and autophagy augments secretory phenotypes. Science.

[34] You JS, Anderson GB, Dooley MS, Hornberger TA (2015). The role of mTOR signaling in the regulation of protein synthesis and muscle mass during immobilization in mice. Dis. Model Mech..

[35] Herranz N (2015). mTOR regulates MAPKAPK2 translation to control the senescence-associated secretory phenotype. Nat. Cell Biol..

[36] Sengupta S, Peterson TR, Laplante M, Oh S, Sabatini DM (2010). mTORC1 controls fasting-induced ketogenesis and its modulation by ageing. Nature.

[37] Jeon SM, Chandel NS, Hay N (2012). AMPK regulates NADPH homeostasis to promote tumour cell survival during energy stress. Nature.

[38] Schmitz FJ, Rosen P, Reinauer H (1995). Improvement of myocardial function and metabolism in diabetic rats by the carnitine palmitoyl transferase inhibitor Etomoxir. Horm. Metab. Res..

[39] Serrano M, Lin AW, McCurrach ME, Beach D, Lowe SW (1997). Oncogenic ras provokes premature cell senescence associated with accumulation of p53 and p16INK4a. Cell.

[40] Terlecki-Zaniewicz L (2018). Small extracellular vesicles and their miRNA cargo are anti-apoptotic members of the senescence-associated secretory phenotype. Aging.

[41] Mashima T, Seimiya H, Tsuruo T (2009). De novo fatty-acid synthesis and related pathways as molecular targets for cancer therapy. Br. J. Cancer.

[42] Chakraborty PK (2015). Role of cystathionine beta synthase in lipid metabolism in ovarian cancer. Oncotarget.

[43] Shi Zhengzheng, Zhou Qing, Gao Shuhong, Li Wenzhi, Li Xin, Liu Zhiming, Jin Pengpeng, Jiang Jie (2019). Silibinin inhibits endometrial carcinoma via blocking pathways of STAT3 activation and SREBP1-mediated lipid accumulation. Life Sciences.

[44] Nacarelli T, Sell C (2017). Targeting metabolism in cellular senescence, a role for intervention. Mol. Cell. Endocrinol..

[45] Dibble CC, Manning BD (2013). Signal integration by mTORC1 coordinates nutrient input with biosynthetic output. Nat. Cell Biol..

[46] Smolina N, Bruton J, Kostareva A, Sejersen T (2017). Assaying mitochondrial respiration as an indicator of cellular metabolism and fitness. Methods Mol. Biol..

[47] Wu Z (2015). Role of p38 mitogen-activated protein kinase in vascular endothelial aging: interaction with Arginase-II and S6K1 signaling pathway. Aging.

[48] Marmisolle I (2017). Reciprocal regulation of acetyl-CoA carboxylase 1 and senescence in human fibroblasts involves oxidant mediated p38 MAPK activation. Arch. Biochem. Biophys..

[49] Wiley CD (2016). Mitochondrial dysfunction induces senescence with a distinct secretory phenotype. Cell. Metab..

[50] Wiley CD (2018). Small-molecule MDM2 antagonists attenuate the senescence-associated secretory phenotype. Sci. Rep..

[51] Niklison-Chirou MV (2017). TAp73 is a marker of glutamine addiction in medulloblastoma. Genes Dev..

[52] Carpintero-Fernandez P, Fafian-Labora J, O’Loghlen A (2017). Technical advances to study extracellular vesicles. Front. Mol. Biosci..

[53] Niklison Chirou M (2008). Microcin J25 induces the opening of the mitochondrial transition pore and cytochrome c release through superoxide generation. FEBS. J..

