# Supplementary material for: FASN activity is important for the initial stages of the induction of senescence
Source: Cell Death Dis. 2019 Apr 8;10(4):318. doi: 10.1038/s41419-019-1550-0 (PMC6453932; doi:10.1038/s41419-019-1550-0)
Supplement: Supplementary file 1 — Figure S1, Figure S2, Figure S3, Figure S4, Figure S5 [file 41419_2019_1550_MOESM1_ESM.pptx]

## Slide 1
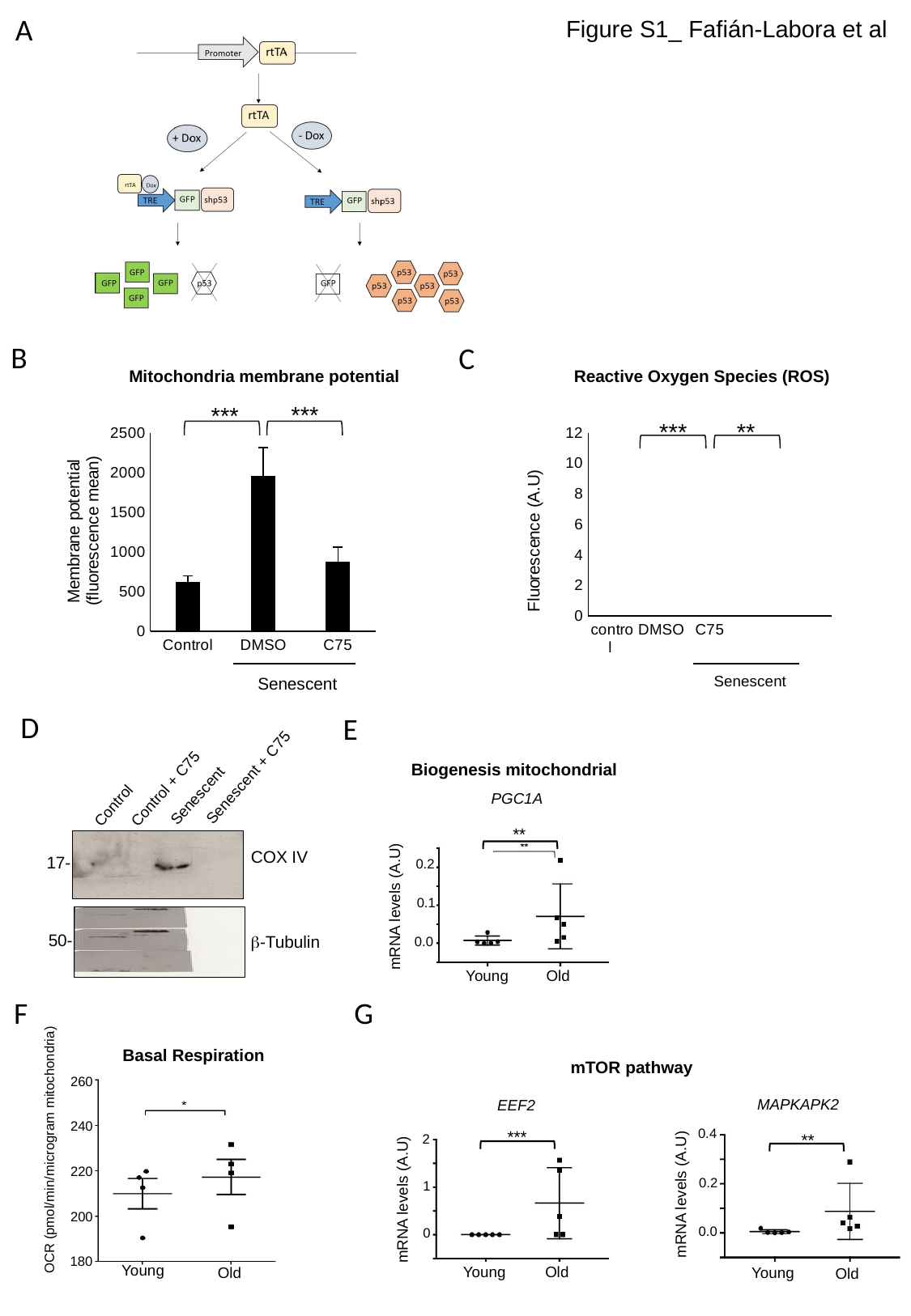

A
Figure S1_ Fafián-Labora et al
B
C
Mitochondria membrane potential
***
***
### Chart
| Category | |
|---|---|
| Control | 615.275 |
| DMSO | 1961.3 |
| C75 | 878.725 |Reactive Oxygen Species (ROS)
***
**
### Chart
| Category | |
|---|---|
| control | 2145.0 |
| DMSO | 4669.66666666667 |
| C75 | 3256.33333333333 |Senescent
Control
COX IV
17-
50-
b-Tubulin
Senescent + C75
Control + C75
Senescent
F
Senescent
D
E
Biogenesis mitochondrial
mTOR pathway
***
**
PGC1A
**
0.2
mRNA levels (A.U)
0.1
0.0
Old
Young
MAPKAPK2
EEF2
0.4
2
mRNA levels (A.U)
0.2
mRNA levels (A.U)
1
0.0
0
Young
Old
Young
Old
Basal Respiration
260
240
220
200
180
Young
Old
OCR (pmol/min/microgram mitochondria)
G

## Slide 2
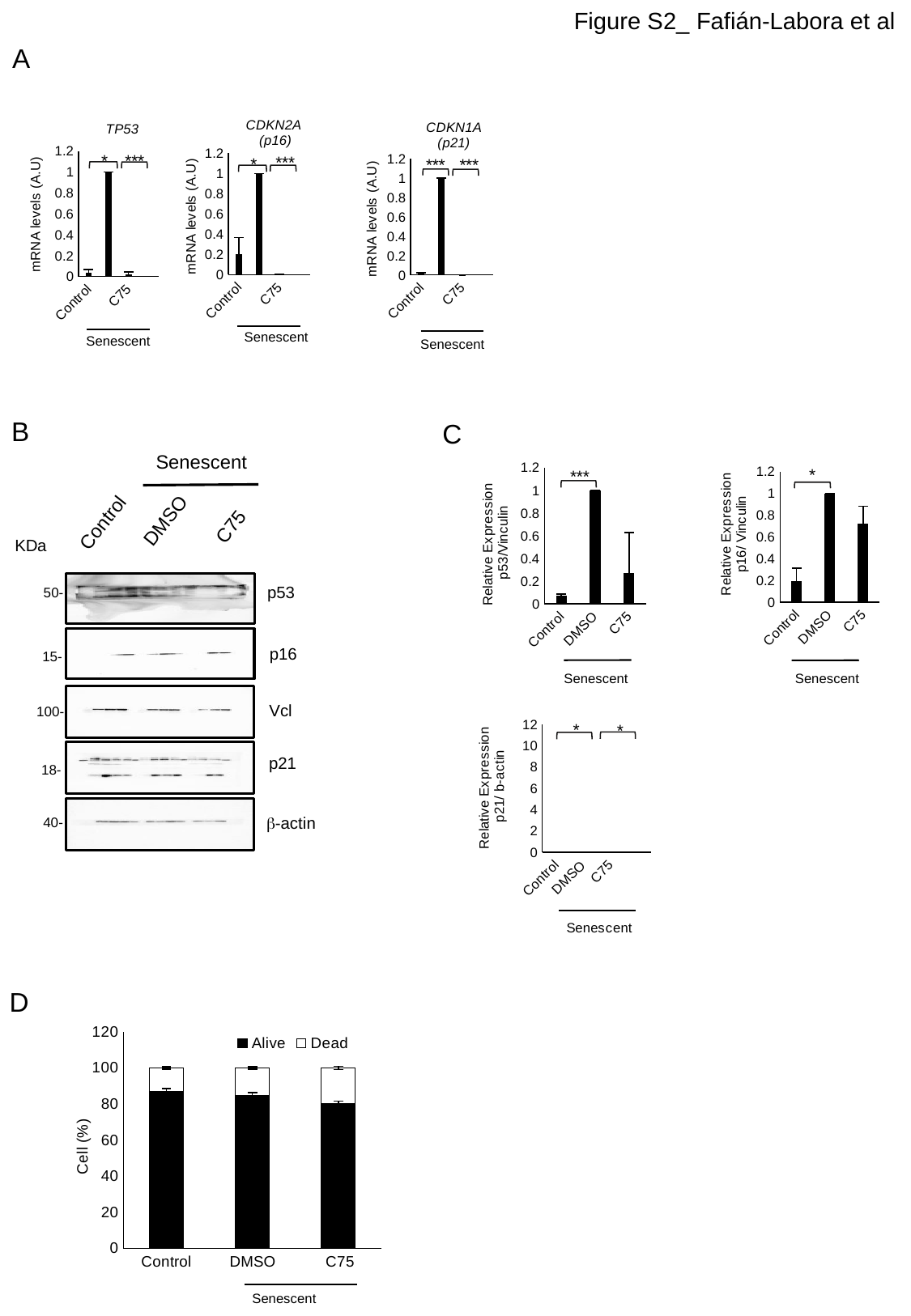

Figure S2_ Fafián-Labora et al
A
[unsupported chart]
Senescent
[unsupported chart]
*
***
*
Senescent
Senescent
[unsupported chart]
### Chart
| Category | Control |
|---|---|
| Control | 0.073622317265634 |
| DMSO | 1.0 |
| C75 | 0.268425544467625 |
### Chart
| Category | Control |
|---|---|
| Control | 0.191974266934527 |
| DMSO | 1.0 |
| C75 | 0.724157657037799 |*
Senescent
Senescent
B
C
Senescent
DMSO
Control
C75
KDa
p53
50-
p16
15-
Vcl
100-
p21
18-
b-actin
40-
### Chart
| Category | Control |
|---|---|
| Control | 0.0566599232904122 |
| DMSO | 1.0 |
| C75 | 0.349773848735073 |D
### Chart
| Category | Alive | Dead |
|---|---|---|
| Control | 86.9 | 13.1 |
| DMSO | 84.7 | 15.3 |
| C75 | 80.1 | 19.90000000000001 |Senescent

## Slide 3
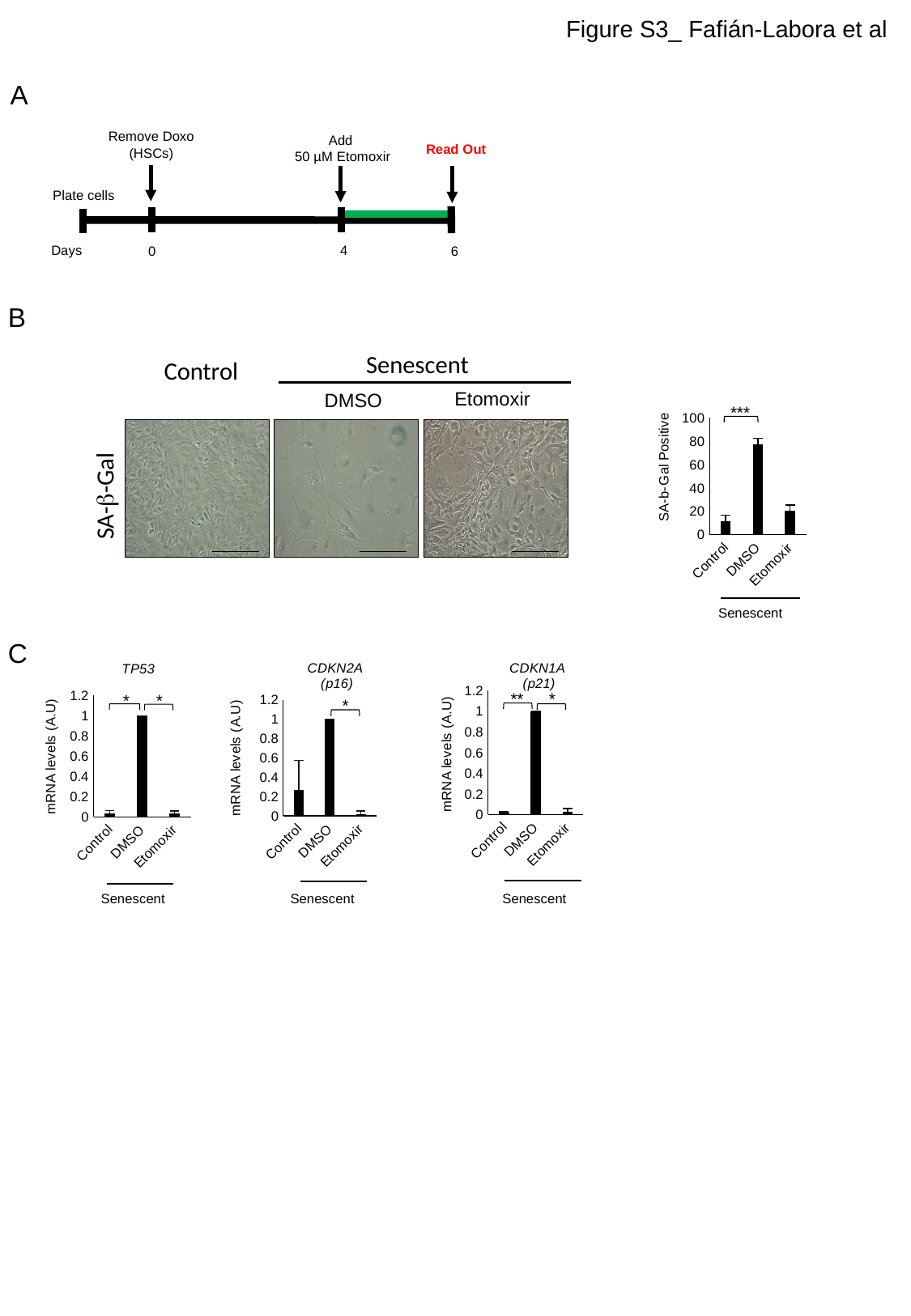

Figure S3_ Fafián-Labora et al
A
Remove Doxo
(HSCs)
Add
50 µM Etomoxir
Plate cells
Days
 4
0
6
Read Out
B
Senescent
Control
Etomoxir
DMSO
### Chart
| Category | |
|---|---|
| Control | 11.77884615384615 |
| DMSO | 77.5 |
| Etomoxir | 20.23035230352302 |
SA-b-Gal
Senescent
***
### Chart: CDKN1A
(p21)
| Category | |
|---|---|
| Control | 0.0154981900722661 |
| DMSO | 1.0 |
| Etomoxir | 0.0273895330780021 |C
### Chart: TP53
| Category | |
|---|---|
| Control | 0.0326537141830165 |
| DMSO | 1.0 |
| Etomoxir | 0.0331548925359922 |
### Chart: CDKN2A
(p16)
| Category | |
|---|---|
| Control | 0.273055934941503 |
| DMSO | 1.0 |
| Etomoxir | 0.0209639622349958 |*
*
Senescent
Senescent
Senescent

## Slide 4
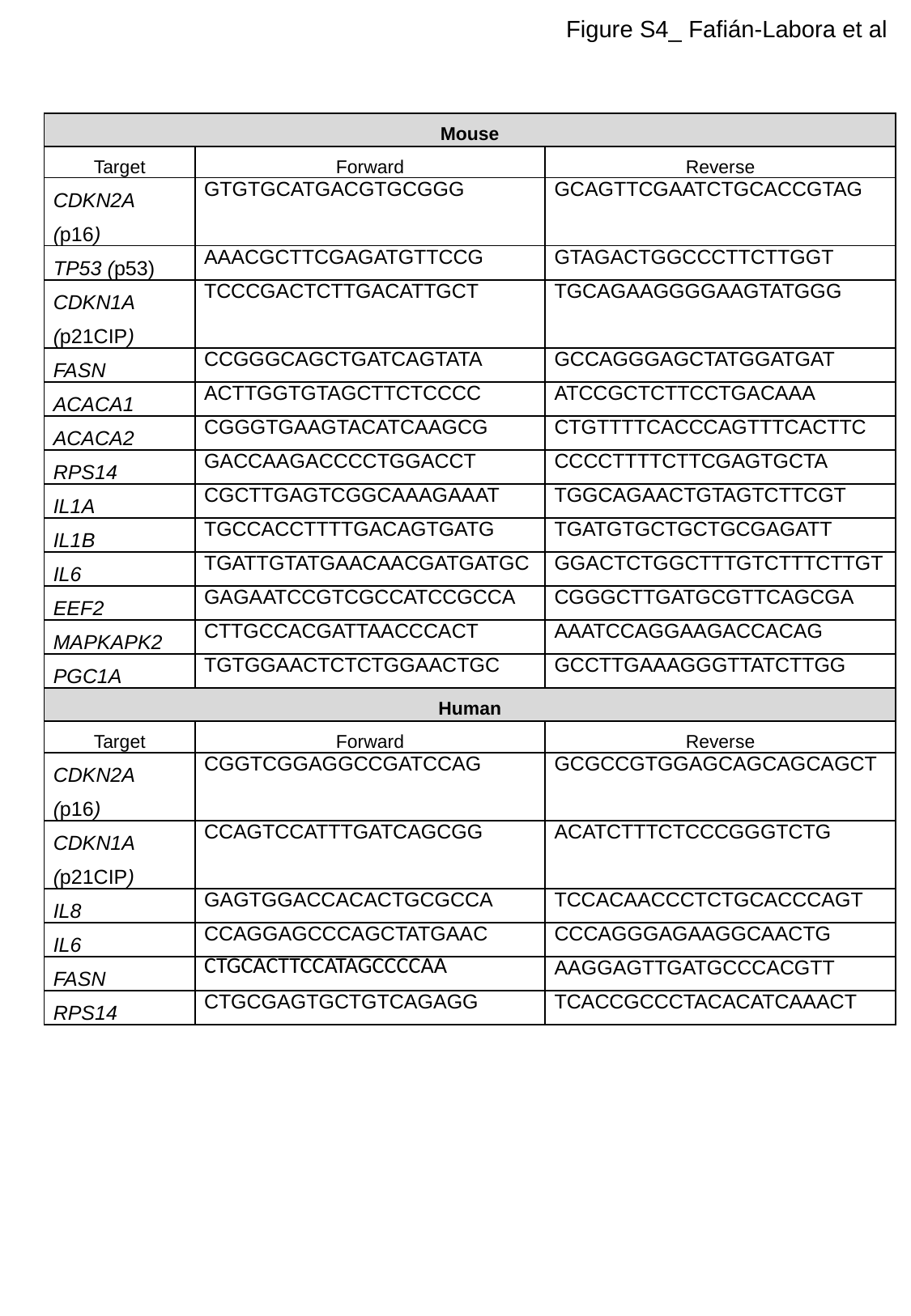

Figure S4_ Fafián-Labora et al
| Mouse | | |
| --- | --- | --- |
| Target | Forward | Reverse |
| CDKN2A (p16) | GTGTGCATGACGTGCGGG | GCAGTTCGAATCTGCACCGTAG |
| TP53 (p53) | AAACGCTTCGAGATGTTCCG | GTAGACTGGCCCTTCTTGGT |
| CDKN1A (p21CIP) | TCCCGACTCTTGACATTGCT | TGCAGAAGGGGAAGTATGGG |
| FASN | CCGGGCAGCTGATCAGTATA | GCCAGGGAGCTATGGATGAT |
| ACACA1 | ACTTGGTGTAGCTTCTCCCC | ATCCGCTCTTCCTGACAAA |
| ACACA2 | CGGGTGAAGTACATCAAGCG | CTGTTTTCACCCAGTTTCACTTC |
| RPS14 | GACCAAGACCCCTGGACCT | CCCCTTTTCTTCGAGTGCTA |
| IL1A | CGCTTGAGTCGGCAAAGAAAT | TGGCAGAACTGTAGTCTTCGT |
| IL1B | TGCCACCTTTTGACAGTGATG | TGATGTGCTGCTGCGAGATT |
| IL6 | TGATTGTATGAACAACGATGATGC | GGACTCTGGCTTTGTCTTTCTTGT |
| EEF2 | GAGAATCCGTCGCCATCCGCCA | CGGGCTTGATGCGTTCAGCGA |
| MAPKAPK2 | CTTGCCACGATTAACCCACT | AAATCCAGGAAGACCACAG |
| PGC1A | TGTGGAACTCTCTGGAACTGC | GCCTTGAAAGGGTTATCTTGG |
| Human | | |
| Target | Forward | Reverse |
| CDKN2A (p16) | CGGTCGGAGGCCGATCCAG | GCGCCGTGGAGCAGCAGCAGCT |
| CDKN1A (p21CIP) | CCAGTCCATTTGATCAGCGG | ACATCTTTCTCCCGGGTCTG |
| IL8 | GAGTGGACCACACTGCGCCA | TCCACAACCCTCTGCACCCAGT |
| IL6 | CCAGGAGCCCAGCTATGAAC | CCCAGGGAGAAGGCAACTG |
| FASN | CTGCACTTCCATAGCCCCAA | AAGGAGTTGATGCCCACGTT |
| RPS14 | CTGCGAGTGCTGTCAGAGG | TCACCGCCCTACACATCAAACT |

## Slide 5
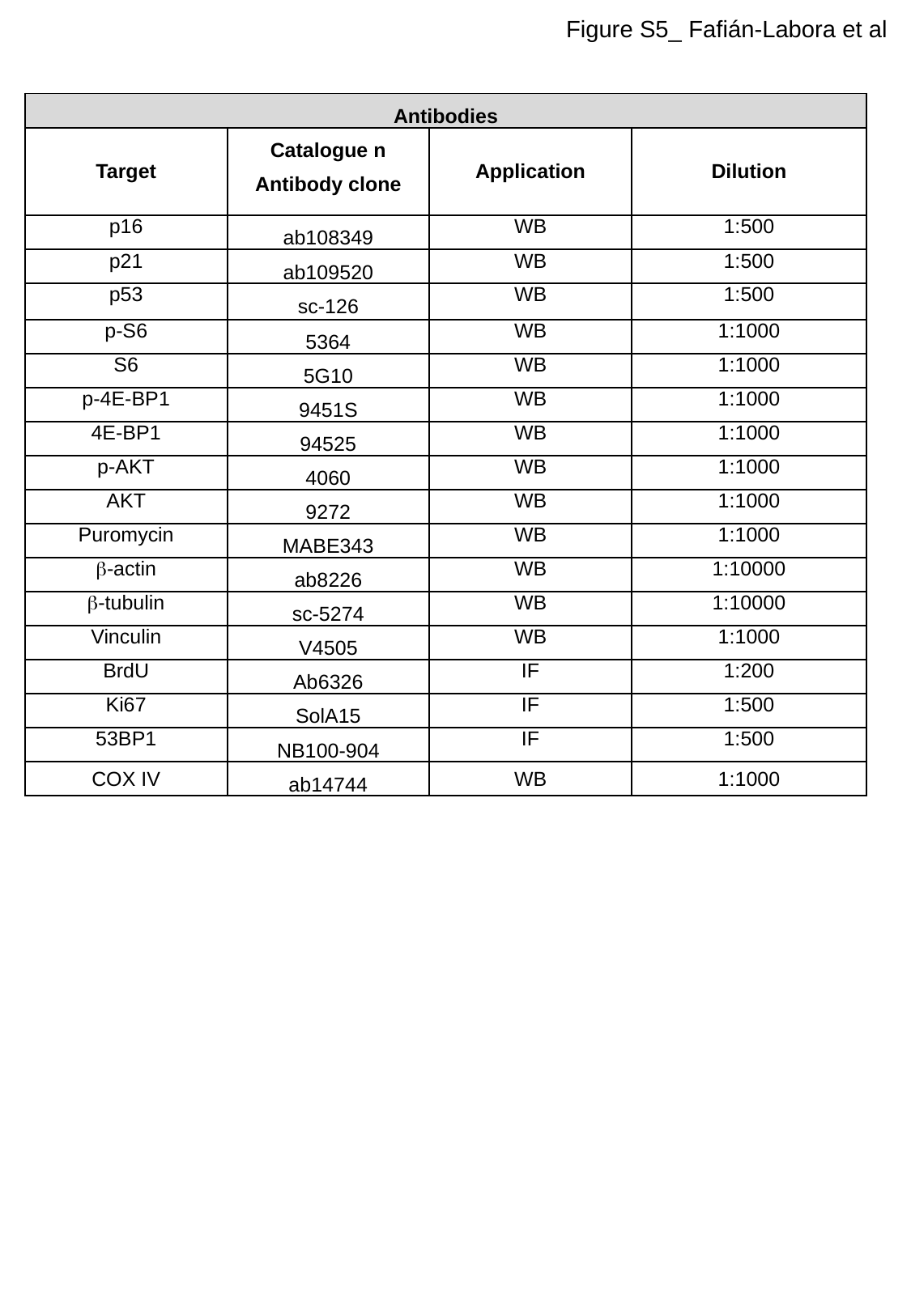

Figure S5_ Fafián-Labora et al
| Antibodies | | | |
| --- | --- | --- | --- |
| Target | Catalogue n Antibody clone | Application | Dilution |
| p16 | ab108349 | WB | 1:500 |
| p21 | ab109520 | WB | 1:500 |
| p53 | sc-126 | WB | 1:500 |
| p-S6 | 5364 | WB | 1:1000 |
| S6 | 5G10 | WB | 1:1000 |
| p-4E-BP1 | 9451S | WB | 1:1000 |
| 4E-BP1 | 94525 | WB | 1:1000 |
| p-AKT | 4060 | WB | 1:1000 |
| AKT | 9272 | WB | 1:1000 |
| Puromycin | MABE343 | WB | 1:1000 |
| b-actin | ab8226 | WB | 1:10000 |
| b-tubulin | sc-5274 | WB | 1:10000 |
| Vinculin | V4505 | WB | 1:1000 |
| BrdU | Ab6326 | IF | 1:200 |
| Ki67 | SolA15 | IF | 1:500 |
| 53BP1 | NB100-904 | IF | 1:500 |
| COX IV | ab14744 | WB | 1:1000 |
