# Supplementary material for: FASN activity is important for the initial stages of the induction of senescence
Source: Cell Death Dis. 2019 Apr 8;10(4):318. doi: 10.1038/s41419-019-1550-0 (PMC6453932; doi:10.1038/s41419-019-1550-0)
Supplement: Supplementary file 2 — suplementary figure legends [file 41419_2019_1550_MOESM2_ESM.docx]

**Supplementary Figure legends**

**Figure S1. C75 inhibits mitochondrial hyperactivation in senescent hepatic stellate cells.** A) Schematic representation of the transgene expressed in the Hepatic Stellate Cells (HSC). The reverse tetracycline-controlled trans-activator (rtTA) protein is regulated by the cytomegalovirus (CMV) promoter; TP53-shRNA is tagged to GFP and controlled by tetracycline response element (TRE) promoter to suppress p53 endogenous expression. Dox: doxycycline, GFP: green fluorescent protein. B) Control, senescent and senescent plus 10μM C75 mouse HSCs cells were labelled with DiOC6(3), a marker of mitochondrial membrane potential. Cells were analysed after 8 days of treatment by flow cytometry and the geometric means were recorded. Data represent the mean±SEM of three independent experiments. Data are represented as geometric means ± SD. ***P<0.0001. C) Level of H2O2 generated in control, senescent and senescent plus 10μM C75 mouse HSCs cells after 8 days of treatment, were measured using the Amplex red hydrogen peroxide assay kit. Data represent the mean±SEM of three independent experiments. (**P<0.001; ***P<0.0001). D) Western blot was performed for COX IV in control, senescent, senescent + 10μM C75. β-tubulin was used as a loading control. E) mRNA levels are shown for PGC1A in liver tissue derived from C57BL/6J female mice from young (4 month) and old (25 month) mice. (**P<0.001). Unpaired two-sided t-test. F) Oxygen composition rate on mitochondria isolated from liver tissue derived from C57BL/6J female mice from young (4 month) and old (25 month) mice. Data represent the mean±SEM of four independent experiments. (*P<0.05). Unpaired two-sided t-test. G) mRNA levels are shown for EEF2 and MAPKAPK2 in liver tissue derived from C57BL/6J female mice from young (4 month) and old (25 month) mice. (**P<0.001; ***P<0.0001). Unpaired two-sided t-test.

**Figure S2. Evaluation of senescence induction in hepatic stellate cells.** A) Relative mRNA levels of the cell cycle inhibitors, TP53, CDKN2A and CDKN1A in control, senescent and senescent plus 10μM C75 mouse HSCs cells. Data represent the mean±SEM of three independent experiments. B) Western blot analysis of the senescent markers (p53, p16 and p21) in cell lysates derived from control, senescent and senescent plus 10μM C75 treatment. β-actin and vinculin (VcI) were used as loading control. Data represent the mean±SEM of two independent experiments. C) Band densitometry quantification for p53, p16 and p21 expression normalized to vinculin or β-actin in mouse HSCs cells. Senescent cells were treated in the presence or absence of 10μM C75. Two-tailed student’s t-test was used to calculate the significance and it was represented as: *P<0.01; ***P<0.0001. D) Apoptosis was measured in control, senescent and senescent plus 10μM C75 mouse HSCs cells. Annexin V-647 and DAPI was detected by flow cytometry analysis. Data represent the mean±SEM of three independent experiments. Data are represented as mean ± SD.

**Figure S3. Etomoxir prevents the induction of cellular senescence.** A) Schematic representation of the experimental settings to determinate the role of FASN pathway in senescent induction of HSCs. After 4 days of senescence induction, 50μM Etomoxir was added to HSCs. B) Representative images and quantification of SA-β-gal in control, senescent and senescent cell treated with 50μM Etomoxir. Scale bar: 100μm. C) Relative mRNA levels of the cell cycle inhibitors, *TP53*, *CDKN2A* and *CDKN1A* in control, senescent and senescent with or without 50μM Etomoxir in HSCs. Data represent the mean±SEM of three independent experiments. Two-tailed student’s t-test was used to calculate the significance and it was represented as: *P< 0.01; **P<0.001; ***P<0.0001.

**Figure S4. List of primer sequences for RT-PCR used in this study.**

**Figure S5. List of antibodies used in this study.**
